# Knowledge, attitude, and practice of healthcare workers on early gastrointestinal cancer in China

**DOI:** 10.3389/fpubh.2023.1191699

**Published:** 2023-07-06

**Authors:** Hui Zhang, Changdong Zhao, Chengwen Song, Youshan Wu, Dongying Wei, Xiuqing Li

**Affiliations:** ^1^Department of Gastroenterology and Hepatology, Lianyungang Second People's Hospital, Lianyungang, China; ^2^Department of Gastroenterology and Hepatology, Affiliated Lianyungang Oriental Hospital of Xuzhou Medical University, Lianyungang, China; ^3^Department of Gastroenterology and Hepatology, The Third People's Hospital of Zhenjiang, Zhenjiang, China

**Keywords:** early gastrointestinal cancer, healthcare workers, knowledge, attitude, practice, cross-sectional study

## Abstract

**Objective:**

Gastrointestinal cancer is the leading cause of cancer-related death in China, and its early screening is largely recommended by healthcare workers. This study investigated the knowledge, attitudes, and practice (KAP) of healthcare workers on early gastrointestinal cancer (EGC).

**Methods:**

This cross-sectional study was conducted on healthcare workers who volunteered to participate from 30 hospitals in China between September and December 2022. A self-administered questionnaire including 37 questions was developed.

**Results:**

A total of 545 completed questionnaires were finally obtained. Healthcare workers had moderate knowledge level [9.22 ± 1.80 (65.88±12.89%), total score: 14], positive attitude [21.84 ± 2.67 (91.01 ± 11.14%), total score: 24], and excellent practice level [19.07 ± 4.43 (79.47 ± 18.44%), total score: 24] on EGC. Pearson's correlation analysis suggested that knowledge score was positively correlated with attitude (*r* = 0.264, *P* < 0.001) and practice score (*r* = 0.140, *P* = 0.001), and higher attitude score was significantly correlated with higher practice score (*r* = 0.380, *P* < 0.001), which were supported and reinforced by structural equation modeling. In addition, subgroup analysis showed that knowledge scores might be influenced by sex, age, education, type of hospital, type of occupation, professional title, and years of working (all *P* < 0.05); attitude scores might be influenced by years of working (*P* < 0.05); and practice scores were statistically distinct among groups of different sex, department, and years of working (all *P* < 0.05).

**Conclusion:**

Healthcare workers have moderate knowledge level, positive attitude, and excellent practice levels on EGC. Good knowledge and positive attitude might be correlated with excellent practice. KAP level might be influenced by sociodemographic characteristics.

## 1. Introduction

Gastrointestinal cancer refers to malignant carcinoma that affects the digestive system, including the gastrointestinal tract and accessory organs. Globally, the age-standardized incidence rate (ASIR) of gastrointestinal cancer was as high as 61.9% per 100,000 person-years in 2019 (1). Common gastrointestinal cancers are recognized as the leading cause of cancer-related death, accounting for 36.2% of overall cancer mortality (2). Based on Cancer Statistics in China 2015, cancers of the esophagus, stomach, colorectum, liver, and pancreas ranked among the top contributors to cancer-related death (3). Under the abovementioned circumstance, efficient intervention could be of priority to palliate the mortality and public health burden of gastrointestinal cancer. The early stage of cancer functions as a critical time window for detection and treatment, during which proper management could largely prolong the survival time of cancer patients (4). Early gastrointestinal cancers (EGC) are limited to the mucosa or submucosa without invading muscularis propria, and timely treatment could greatly exert positive effects on a 5-year survival rate and prognosis (5, 6).

A series of screening methods were available for the detection of EGC, such as barium-meal photofluorography, endoscopic examination, and serological biomarker testing. Despite the increasing number of individuals involved in the screening of gastrointestinal cancer, the population coverage remains to be elevated and the early detection rate is still unsatisfactory (7). Deficiency in clinicians and medical facilities, imbalance in socioeconomic development, and inadequate public awareness of gastrointestinal cancer screening could to some extent account for such phenomenon. In addition, individuals might be reluctant to undergo screening because of insufficient knowledge, the invasiveness of examination, physical discomfort, and emotional concerns (8). Therefore, promoting the screening rate would be urgently needed for the better prevention and control of EGC.

In China, individual screening of gastrointestinal cancer is largely carried out according to the healthcare workers' suggestions during routine consultations (7). In addition, the knowledge and attitude of healthcare workers toward gastrointestinal cancer could determine their prescription process, which further impacts the individual involvement in screening. Therefore, a better understanding of knowledge, attitude, and practice (KAP) among healthcare workers is necessary to formulate feasible strategies and perform effective intervention programs. Previous literature suggested that there was a need for improvement in the KAP regarding cancer diagnosis and treatment. For example, although healthcare workers were aware of the importance of cervical cancer prevention, the attitudes and practices toward screening were negative (9). Furthermore, while nurses had sufficient knowledge about breast cancer, they still required additional information on cancer risk estimation (10). However, there is a dearth of KAP for gastrointestinal cancer among healthcare workers in China to date. Additionally, sociodemographic factors might serve as modifiers of KAP toward cancers, however, which remained undetermined on the KAP related to gastrointestinal cancer (11). In this study, we aimed to provide a reference of Chinese healthcare workers' KAP of gastrointestinal cancer and related sociodemographic factors.

## 2. Materials and methods

### 2.1. Study design and participants

This cross-sectional study was conducted from September to December 2022 and included healthcare workers from 30 hospitals in Jiangsu Province, aiming to enhance the generalizability of the findings. A simple random sampling method was used to select healthcare workers from each hospital who volunteered to participate in the study. The hospital's information is shown in Supplementary Table 2. This study was approved by the institutional ethics committee of The Second People's Hospital of Lianyungang City in Jiangsu Province, and the informed consent of the participants was obtained.

### 2.2. Questionnaire

The questionnaire was designed according to guidelines, including the *China Guideline for Screening, Early Detection and Early Treatment of Gastric Cancer* (2022, Beijing), *China Guideline for Screening, Early Detection and Early Treatment of Esophageal Cancer* (2022, Beijing), and *Guideline for the Diagnosis and Treatment of Esophageal Cancer* (2022 Edition). Then, the questionnaire was modified according to the suggestions of five experts, and then, the pilot survey was performed on a small scale (48 questionnaires were dispatched).

The final questionnaire was a Chinese version that collected data from 37 questions in four dimensions (Supplementary Table 1). Specifically, the sociodemographic characteristics dimension consisted of 10 questions, the knowledge dimension included 14 questions, the attitude dimension included 7 questions, and the practice dimension included 6 questions. Every correct answer in the knowledge dimension was scored by 1 point, and a wrong or unclear answer was scored by 0 point, ranging from 0 to 14 points. For attitude and practice dimensions, the answers were scored from 0 to 4 points, and the range of the total score was provided (0 to 24); the seventh question was not included because of unable to be scored and described only. The knowledge dimension of our study encompassed a comprehensive understanding of gastrointestinal cancer, including its definition (items 1–5), risk factors (items 6–7), early detection (item 8), treatment options (items 9–13), and prevention (item 14). Participants' attitudes toward gastrointestinal cancer were assessed to gauge their perspectives (items 2 and 3), beliefs (items 1, 5), and emotional responses (items 4 and 6) toward this disease. Additionally, participants' practices were examined to evaluate their behaviors and actions concerning the prevention (items 1–3), diagnosis (item 4), and treatment (items 5 and 6) of gastrointestinal cancer.

### 2.3. Procedures

The online questionnaire based on the SoJump application of WeChat was used for the survey, and a QR code was generated to allow the data collection through WeChat. Participants logged in by scanning the QR code sent by WeChat and then completed the questionnaire. To guarantee the quality and completeness of the questionnaire survey, each IP address could only submit the questionnaire once, and all questions in the questionnaire were mandatory. The completeness, internal coherence, and reasonableness of all questionnaires were checked by the investigators.

### 2.4. Statistical analysis

Stata 17.0 (Stata Corporation, College Station, TX, USA) software was used for the statistical analysis. A descriptive analysis of the general characteristics of the participants and the KAP scores was performed as follows: Continuous variables in a normal distribution (KAP scores) were described by mean ± standard deviation (SD), and the maximum and minimum values were also reported. The hundred-mark system (HMS) of the KAP score was calculated by the following formula: sum of the knowledge score—minimum possible score)/range of the knowledge score. Categorical data including the demographic characteristics and answers to different questions were described by *n* (%). For the knowledge dimension, the HMS score of ≤50% was defined as “low knowledge level,” 50–85% was defined as “moderate knowledge level,” and >85% is defined as “high knowledge level.” For the attitude dimension, the HMS score <30% indicates “negative attitude,” 30%−60% indicates “neutral attitude,” and >60% indicates “positive attitude.” For the practice dimension, the HMS score <25% is defined as “poor practice,” 25%−50% is defined as “general practice,” 51%−75% is defined as “good practice,” and >75% is defined as “excellent practice.” The data's normality was evaluated through Kolmogorov–Smirnov test. When the data followed a normal distribution, the comparison between the two groups was conducted using Student's *t*-test, while analysis of variance (ANOVA) was employed for comparisons among multiple groups. In cases where the data exhibited a skewed distribution, the Mann–Whitney *U* test was utilized for comparing two groups, and the Kruskal–Wallis analysis of variance was employed for comparing multiple groups. Pearson correlation analysis was used to assess the correlation in the three dimensions. The differences in the knowledge, attitude, and practice of EGC were compared in healthcare workers with different general characteristics. Structural equation modeling (SEM) analysis was further conducted to explore the direct and indirect associations between sociodemographic characteristics and KAP scores. A two-sided *P*-value of <0.05 was considered statistically significant.

## 3. Results

The pilot survey findings (*N* = 48) demonstrated high internal consistency in the KAP dimensions, with Cronbach's α values of 0.896, 0.821, and 0.902, respectively. The Kaiser–Meyer–Olkin (KMO) measure for the KAP dimensions was 0.803, 0.841, and 0.889, respectively. The validity and reliability of the questionnaire were assessed, revealing a Cronbach's α of 0.873 and a KMO of 0.720. As shown in Supplementary Table 3, strong reliability and adequate internal consistency measures in the design of the questionnaire were observed. The results from Bartlett's test and the KMO values provided support for conducting factor analysis, indicating robust construct validity.

A total number of 545 questionnaires were collected for analysis. The majority of participants (66.98%) were between 31 and 50 years old, and most subjects were women (72.48%) and obtained educational attainments of technical secondary school or junior college (80.00%). Participants working in tertiary public hospitals accounted for 70.28%, and nearly half of the whole population (41.83%) were affiliated to the internal medicine department. The occupation of participants was mainly doctor (42.86%) and nurse (48.19%), and 64.95% of them owned the professional title of medium or above. Years of working were mainly distributed in ≥11 years (62.93%). Although upper gastrointestinal disease was reported in more than half of participants or their family and friends (57.25%), gastrointestinal cancer (such as esophageal or gastric cancer) occurred uncommonly among participants' families (11.19%) (Table 1).

**Table 1:** Baseline characteristics of the participants.

**Characteristics**	***N* (%)**	**Knowledge, mean** ±**SD**			**Attitude, mean** ±**SD**			**Practice, mean** ±**SD**		
		**Score**	**HMS**	***P*** **-value**	**Score**	**HMS**	***P*** **-value**	**Score**	**HMS**	***P*** **-value**
**Total**	545 (100)	9.22 ± 1.80	65.88 ± 12.89		21.84 ± 2.67	91.01 ± 11.14		19.07 ± 4.43	79.47 ± 18.44	
**Sex**				0.018			0.823			0.030
Male	150 (27.52)	9.46 ± 1.71	67.57 ± 12.21		21.71 ± 3.10	90.47 ± 12.90		19.71 ± 4.34	82.11 ± 18.09	
Female	395 (72.48)	9.13 ± 1.83	65.24 ± 13.09		21.89 ± 2.50	91.21 ± 10.41		18.83 ± 4.44	78.47 ± 18.51	
**Age (years)**				<0.001			0.075			0.053
<30	131 (24.04)	8.64 ± 2.01	61.72 ± 14.37		21.27 ± 3.31	88.65 ± 13.79		18.76 ± 4.53	78.15 ± 18.89	
31–40	208 (38.17)	9.38 ± 1.74	66.96 ± 12.45		22.25 ± 2.16	92.69 ± 8.98		19.70 ± 4.29	82.09 ± 17.88	
41–50	157 (28.81)	9.51 ± 1.70	67.93 ± 12.12		21.80 ± 2.74	90.84 ± 11.42		18.63 ± 4.63	77.63 ± 19.30	
>50	49 (8.99)	9.22 ± 1.48	65.89 ± 10.54		21.78 ± 2.31	90.73 ± 9.63		18.67 ± 3.82	77.81 ± 15.92	
**Education**				0.004			0.724			0.335
Technical secondary school/junior college	436 (80.00)	9.12 ± 1.86	65.17 ± 13.27		21.79 ± 2.77	90.81 ± 11.54		19.15 ± 4.44	79.80 ± 18.51	
College or above	109 (20.00)	9.62 ± 1.51	68.74 ± 10.81		22.04 ± 2.25	91.82 ± 9.37		18.76 ± 4.37	78.17 ± 18.21	
**Type of hospital**				0.013			0.054			0.060
Primary public hospital	79 (14.50)	8.92 ± 2.19	63.74 ± 15.67		21.43 ± 3.33	89.29 ± 13.88		19.75 ± 4.37	82.28 ± 18.22	
Secondary public hospital	32 (5.87)	9.53 ± 2.46	68.08 ± 17.59		20.94 ± 4.33	87.24 ± 18.05		17.19 ± 5.87	71.61 ± 24.44	
Tertiary public hospital	383 (70.28)	9.36 ± 1.54	66.84 ± 11.00		22.08 ± 2.32	91.98 ± 9.68		19.20 ± 4.23	80.00 ± 17.62	
Private hospital	51 (9.36)	8.49 ± 2.27	60.64 ± 16.19		21.29 ± 2.48	88.73 ± 10.32		18.25 ± 4.66	76.06 ± 19.40	
**Type of occupation**				<0.001			0.352			0.091
Doctor	225 (42.86)	9.60 ± 1.53	68.54 ± 10.95		22.08 ± 2.45	91.98 ± 10.21		19.43 ± 4.50	80.96 ± 18.76	
Nurse	253 (48.19)	9.02 ± 1.84	64.43 ± 13.16		21.77 ± 2.72	90.69 ± 11.35		19.13 ± 4.17	79.69 ± 17.36	
Others	47 (8.95)	8.89 ± 2.00	63.53 ± 14.30		21.51 ± 3.19	89.63 ± 13.29		17.96 ± 4.70	74.82 ± 19.58	
**Professional title**				<0.001			0.152			0.749
None	48 (8.81)	8.21 ± 2.41	58.63 ± 17.18		21.04 ± 4.39	87.67 ± 18.29		17.92 ± 5.63	74.65 ± 23.44	
Junior	143 (26.24)	8.84 ± 1.96	63.14 ± 14.02		21.54 ± 2.72	89.74 ± 11.33		19.36 ± 4.14	80.65 ± 17.25	
Medium	206 (37.80)	9.42 ± 1.71	67.30 ± 12.24		21.95 ± 2.35	91.44 ± 9.78		19.23 ± 4.30	80.12 ± 17.91	
Vice-senior	113 (20.73)	9.66 ± 1.29	69.03 ± 9.19		22.21 ± 2.30	92.55 ± 9.57		18.89 ± 4.39	78.72 ± 18.31	
Senior	35 (6.42)	9.60 ± 1.31	68.57 ± 9.36		22.37 ± 2.06	9.32 ± 8.58		19.17 ± 4.57	79.88 ± 19.05	
**Years of working (years)**				<0.001			0.028			0.029
≤ 5	108 (19.82)	8.56 ± 2.09	61.18 ± 14.95		21.17 ± 3.42	88.19 ± 14.25		18.51 ± 4.80	77.12 ± 19.98	
5–10	94 (17.25)	9.32 ± 2.06	66.56 ± 14.75		22.01 ± 2.38	91.71 ± 9.90		20.05 ± 4.11	83.55 ± 17.12	
11–15	139 (25.50)	9.37 ± 1.59	66.96 ± 11.35		22.25 ± 2.49	92.72 ± 10.38		19.40 ± 4.46	80.85 ± 18.58	
≥16	204 (37.43)	9.43 ± 1.57	67.33 ± 11.19		21.84 ± 2.41	91.01 ± 10.06		18.70 ± 4.28	77.90 ± 17.83	
**Department**				0.096			0.694			0.014
Internal medicine department	228 (41.83)	9.46 ± 1.72	67.58 ± 12.26		21.98 ± 2.54	91.58 ± 10.59		19.73 ± 4.08	82.20 ± 17.00	
Surgery department	90 (16.51)	9.03 ± 1.86	64.52 ± 13.31		21.57 ± 3.41	89.86 ± 14.22		19.39 ± 4.14	80.79 ± 17.24	
Oncology department	12 (2.20)	9.17 ± 1.03	65.48 ± 7.36		22.50 ± 1.78	93.75 ± 7.43		19.00 ± 3.64	79.17 ± 15.18	
Other departments	215 (39.45)	9.06 ± 1.88	64.68 ± 13.45		21.78 ± 2.50	90.74 ± 10.41		18.25 ± 4.82	76.05 ± 20.07	
**Upper gastrointestinal diseases, such as chronic gastritis, reflux esophagitis, or gastric ulcer in the participants, the family, or friends**				0.072			0.105			0.987
Yes	312 (57.25)	9.41 ± 1.48	67.22 ± 10.57		22.05 ± 2.46	91.88 ± 10.25		19.09 ± 4.42	79.53 ± 18.41	
No	233 (42.75)	8.97 ± 2.14	64.10 ± 15.30		21.56 ± 2.92	89.84 ± 12.16		19.06 ± 4.45	79.40 ± 18.54	
**Esophageal or gastric cancer in the family**				0.681			0.594			0.550
Yes	61 (11.19)	9.39 ± 1.28	67.10 ± 9.15		22.10 ± 2.26	92.08 ± 9.43		19.39 ± 4.35	80.81 ± 18.13	
No	484 (88.81)	9.20 ± 1.86	65.73 ± 13.28		21.81 ± 2.72	90.87 ± 11.34		19.03 ± 4.44	79.30 ± 18.50	

HMS, hundred-mark system.

The mean knowledge, attitude, and practice score was 9.22 ± 1.80 (65.88±12.89%, total score: 14), 21.84 ± 2.67 (91.01±11.14%, total score: 24), and 19.07 ± 4.43 (79.47±18.44%, total score: 24) among all respondents. In addition, subgroup analysis revealed that knowledge scores differed significantly among groups of different sex, age, education, type of hospital, type of occupation, professional title, and years of working (all *P* < 0.05); attitude scores were distinctly disparate among groups of different years of working (*P* < 0.05) (those with long years of working, such as 11–15 years, had more positive attitude); and practice scores were statistically distinct among groups of different sex, department, and years of working (all *P* < 0.05) (Table 1).

The correct rate of knowledge dimension ranged from 6.97% to 96.33%. Most participants (96.33%) agreed that to prevent EGC, the following items should be paid additional attention to daily living: appropriate dietary structure, personal hygiene, regular diet, avoiding heavy smoking or drinking, and defecating every day. However, only 6.97% of them knew that EGC is mainly treated endoscopically, including endoscopic mucosal resection (EMR) and endoscopic submucosal dissection (ESD), of which EMR is more frequently used (Table 2). Based on the cutoff value, as high as 86.24% of respondents were assigned to a group of moderate knowledge level.

**Table 2:** Knowledge dimension of the participants.

**Knowledge**	**Correct *N* (%)**	**Wrong *N* (%)**	**Unclear *N* (%)**
1. The digestive tract includes the oral cavity, oropharynx, esophagus, stomach, duodenum, small intestine, colon, and rectum	509 (93.39)	21 (3.85)	15 (2.75)
2. Early esophageal cancer refers to esophageal cancer with an invasion depth of reaching the mucosal layer, but not accompanied by lymph node metastasis	459 (84.22)	43 (7.89)	43 (7.89)
3. Early gastric cancer refers to the tumors that the invasion is limited to the mucosal or submucosal layer and is related to the lesion size and lymph node metastasis	111 (20.37)	390 (71.56)	44 (8.07)
4. Early colorectal cancer refers to the disease that the cancerous tissue restricted to the mucosal and submucosal layer, regardless of lymph node metastasis	321 (58.90)	168 (30.83)	56 (10.28)
5. Early gastrointestinal cancer refers to early tumors of the digestive tract; most digestive tract cancers have no specific symptoms in the early stage and thus are easy to be ignored	507 (93.03)	17 (3.12)	21 (3.85)
6. Individuals aged >40 years old, with a history of precancerous lesions of the digestive tract, family history of digestive tract tumor, evident gastrointestinal symptoms, prefer salty, fried, or smoked food (>3 meals/week), and/or heavy smoking and alcohol drinking are considered high-risk population	523 (95.96)	8 (1.47)	14 (2.57)
7. The first and foremost task of early gastrointestinal cancer screening is to identify the high-risk population	521 (95.60)	13 (2.39)	11 (2.02)
8. Currently, tumor marker detection, such as CEA and CA125, is the major screening method	81 (14.86)	430 (78.90)	34 (6.24)
9. Endoscopy can identify the lesions in the digestive tract, but cannot obtain biopsy of the cancerous sites for pathological examination	328 (60.18)	192 (35.23)	25 (4.59)
10. Early gastrointestinal cancer can be completely removed under endoscopy, while no chemotherapy is required after surgery, and the 5-year survival rate is >90%	111 (20.37)	381 (69.91)	53 (9.72)
11. Compared with regular endoscopy, precision endoscopy can provide the examinations of magnifying endoscopy, staining endoscopy, and electronic staining endoscopy, which makes the lesion examination more subtle, therefore help determining whether the lesion is cancerous, as well as the range and depth of invasion, and differentiation degree, and evaluate the presence of indications for microscopic treatment	500 (92.74)	8 (1.47)	37 (6.79)
12. Compared with traditional endoscopy, the emerging endoscopic narrow band imaging (NBI) in recent years allows us not only to accurately observe the morphology of the mucosal epithelium of the digestive tract but also observe the morphology of the epithelial vascular network, thereby improving the accuracy of endoscopic diagnosis	493 (90.46)	5 (0.92)	47 (8.62)
13. Early gastrointestinal cancer is mainly treated endoscopically, including endoscopic mucosal resection (EMR) and endoscopic submucosal dissection (ESD), of which EMR is more frequently used. EMR can be used for patients with early stages of esophageal cancer, gastric cancer, or colorectal cancer	38 (6.97)	455 (83.49)	52 (9.54)
14. To prevent early gastrointestinal cancer, the following items should be paid with additional attentions in daily living: appropriate dietary structure, personal hygiene, regular diet, avoiding heavy smoking or drinking, and defecating every day	525 (96.33)	8 (1.47)	12 (2.20)

The majority of participants held the answers of “Highly agree” or “Agree” in the attitude dimension, with a proportion from 82.75% to 99.08%. Most respondents (99.08%) would be willing to popularize the knowledge of EGC. However, subtly less population (82.75%) held positive attitude toward that esophageal cancer and stomach cancer can be radically treated or completely cured (Table 3). Overall, 99.27% of participants owned a good attitude level. Additionally, the majority of participants agreed that unhealthy eating habits and lifestyle, gastrointestinal disease, and family history were associated with gastrointestinal cancer (Table 4).

**Table 3:** Attitude dimension of the participants.

**Attitude**	**Highly agree *N* (%)**	**Agree *N* (%)**	**Neutral *N* (%)**	**Disagree *N* (%)**	**Highly disagree *N* (%)**
A1. You think high-risk population, regardless of whether they have symptoms or not, should receive the screen for early gastrointestinal cancer	456 (83.67)	73 (13.39)	9 (1.65)	4 (0.73)	3 (0.55)
A2. You think that early esophageal cancer and stomach cancer can be radically treated (completely cured).	304 (55.78)	147 (26.97)	56 (10.28)	29 (5.32)	(1.65)
A3. You think that changing the lifestyle can prevent the occurrence of digestive tract cancer	318 (58.35)	187 (34.31)	32 (5.87)	6 (1.10)	2 (0.37)
A4. You think that the general people's awareness of early gastrointestinal cancer is far from enough, and it is necessary to increase the intensity of health education	422 (77.43)	117 (21.47)	2 (0.37)	3 (0.55)	1 (0.18)
A5. You think that popularization of science in general people is of great significance for the reduction of the occurrence and development of early gastrointestinal cancer	431 (79.08)	103 (18.90)	8 (1.47)	2 (0.37)	1 (0.18)
A6. You are willing to do your best to popularize the knowledge of early gastrointestinal cancer	416 (76.33)	124 (22.75)	2 (0.37)	1 (0.18)	2 (0.37)

**Table 4:** The detailed answers to the question “A7. Which of the followings do you consider to be risk factors of gastrointestinal cancer?”

**Risk factor**	**0, *N* (%)**	**1, *N* (%)**	**2, *N* (%)**	**3, *N* (%)**	**4, *N* (%)**	**5, *N* (%)**	**6, *N* (%)**	**7, *N* (%)**	**8, *N* (%)**	**9, *N* (%)**	**10, *N* (%)**
Female	68 (12.48)	47 (8.62)	42 (7.71)	60 (11.01)	41 (7.52)	129 (23.67)	37 (6.79)	29 (5.32)	40 (7.34)	18 (3.30)	34 (6.24)
Male	43 (7.89)	24 (4.40)	22 (4.04)	35 (6.42)	27 (4.95)	102 (18.72)	68 (12.48)	73 (13.39)	69 (12.66)	35 (6.42)	47 (8.62)
Family history of esophageal or gastric cancer	17 (3.12)	9 (1.65)	12 (2.20)	18 (3.30)	14 (2.57)	52 (9.54)	36 (6.61)	84 (15.41)	89 (16.33)	76 (13.94)	138 (25.32)
Long-term smoking	14 (2.57)	7 (1.28)	8 (1.47)	30 (5.50)	22 (4.04)	69 (12.66)	46 (8.44)	74 (13.58)	102 (18.72)	61 (11.19)	112 (20.55)
Long-term heavy alcohol drinking	14 (2.57)	5 (0.92)	13 (2.39)	19 (3.49)	7 (1.28)	47 (8.62)	46 (8.44)	66 (12.11)	111 (20.37)	76 (13.94)	141 (25.87)
High-salt diet	8 (1.47)	14 (2.57)	11 (2.02)	25 (4.59)	22 (4.04)	68 (12.48)	77 (14.13)	68 (12.48)	95 (17.43)	46 (8.44)	111 (20.37)
Spicy diet	11 (2.02)	9 (1.65)	11 (2.02)	21 (3.85)	23 (4.22)	67 (12.29)	82 (15.05)	61 (11.19)	95 (17.43)	61 (11.19)	104 (19.08)
Oily diet	11 (2.02)	10 (1.83)	9 (1.65)	22 (4.04)	30 (5.50)	69 (12.66)	82 (15.05)	70 (12.84)	101 (18.53)	51 (9.36)	90 (16.51)
Eat pickled, smoked, fried, or deep-fried food often	8 (1.47)	10 (1.83)	8 (1.47)	11 (2.02)	17 (3.12)	41 (7.52)	45 (8.26)	85 (15.60)	105 (19.27)	76 (13.94)	139 (25.50)
Eat vegetables or fruits often	247 (45.32)	57 (10.46)	61 (11.19)	28 (5.14)	14 (2.57)	26 (4.77)	26 (4.77)	19 (3.49)	27 (4.95)	15 (2.75)	25 (4.59)
Eat processed meat or sausages often	8 (1.47)	12 (2.20)	14 (2.57)	31 (5.69)	18 (3.30)	80 (14.68)	70 (12.84)	77 (14.13)	99 (18.17)	49 (8.99)	87 (15.96)
Eat leftovers often	7 (1.28)	13 (2.39)	9 (1.65)	20 (3.67)	27 (4.95)	73 (13.39)	70 (12.84)	82 (15.05)	103 (18.90)	53 (9.72)	88 (16.15)
Usually eat a hot meal and drink hot water or hot tea	8 (1.47)	10 (1.83)	13 (2.39)	16 (2.94)	16 (2.94)	47 (8.62)	57 (10.46)	83 (15.23)	92 (16.88)	80 (14.68)	123 (22.57)
Irregular diet	7 (1.28)	10 (1.83)	7 (1.28)	24 (4.40)	20 (3.67)	61 (11.19)	60 (11.01)	80 (14.68)	96 (17.61)	76 (13.94)	104 (19.08)
Eat very fast	11 (2.02)	13 (2.39)	17 (3.12)	30 (5.50)	36 (6.61)	75 (13.76)	75 (13.76)	89 (16.33)	90 (16.51)	40 (7.34)	69 (12.66)
Overeating	16 (1.83)	10 (1.83)	8 (1.47)	26 (4.77)	25 (4.59)	62 (11.38)	66 (12.11)	83 (15.23)	106 (19.45)	62 (11.38)	87 (15.96)
Obesity	15 (2.75)	10 (1.83)	12 (2.20)	18 (3.30)	33 (6.06)	60 (11.01)	92 (16.88)	87 (15.96)	105 (19.27)	52 (9.54)	61 (11.19)
Lack of exercise	14 (2.57)	18 (3.30)	33 (6.06)	29 (5.32)	38 (6.97)	86 (15.78)	86 (15.78)	81 (14.86)	79 (14.50)	30 (5.50)	51 (9.36)
With the fast pace of life and being stressful	5 (0.92)	12 (2.20)	11 (2.02)	23 (4.22)	31 (5.69)	68 (12.48)	68 (12.48)	95 (17.43)	111 (20.37)	50 (9.17)	71 (13.03)
Being sulking and in a depressive mood often	6 (1.10)	10 (1.83)	11 (2.02)	23 (4.22)	22 (4.04)	66 (12.11)	64 (11.74)	81 (14.86)	115 (21.10)	61 (11.19)	86 (15.78)
Air pollution	11 (2.02)	19 (3.49)	21 (3.85)	35 (6.42)	26 (4.77)	79 (14.50)	71 (13.03)	87 (15.96)	90 (16.51)	42 (7.71)	64 (11.74)
Helicobacter pylori infection	6 (1.10)	12 (2.20)	6 (1.10)	17 (3.12)	18 (3.30)	38 (6.97)	62 (11.38)	58 (10.64)	102 (18.72)	82 (15.05)	144 (26.42)
Chronic esophagitis	7 (1.28)	11 (2.02)	7 (1.28)	19 (3.49)	25 (4.59)	61 (11.19)	79 (14.50)	67 (12.29)	115 (21.10)	59 (10.83)	95 (17.43)
Gastroesophageal reflux disease	8 (1.47)	6 (1.10)	9 (1.65)	22 (4.04)	30 (5.50)	61 (11.19)	69 (12.66)	72 (13.21)	110 (20.18)	59 (10.83)	99 (18.17)
Esophageal ulcer	9 (1.65)	8 (1.47)	5 (0.92)	18 (3.30)	20 (3.67)	43 (7.89)	61 (11.19)	70 (12.84)	120 (22.02)	69 (12.66)	122 (22.39)
Chronic gastritis	9 (1.65)	8 (1.47)	8 (1.47)	34 (6.24)	32 (5.87)	78 (14.31)	73 (13.39)	61 (11.19)	104 (19.08)	53 (9.72)	85 (15.60)
Gastric ulcer	10 (1.83)	8 (1.47)	7 (1.28)	20 (3.67)	19 (3.49)	45 (8.26)	62 (11.38)	69 (12.66)	124 (22.75)	67 (12.29)	114 (20.92)
Gastric polyp	10 (1.83)	8 (1.47)	11 (2.02)	28 (5.14)	13 (2.39)	63 (11.56)	74 (13.58)	64 (11.74)	98 (17.98)	79 (14.50)	97 (17.80)
Post-gastric surgery	15 (2.75)	7 (1.28)	9 (1.65)	25 (4.59)	24 (4.40)	67 (12.29)	73 (13.39)	73 (13.39)	109 (20.00)	60 (11.01)	83 (15.23)

(0, no risk, 1–10, gradually increased risk, and 10, extremely high risk).

More than half of the whole population chose “Always” or “Often” in the practice dimension (Table 5). Most subjects (93.77%) gave positive responses toward propagating knowledge of EGC to patients. In addition, the lowest frequency of participants (67.71%) would actively participate in the popularization of knowledge on the importance of EGC screening as possible. The proportion of respondents with excellent practice ranked at the top (58.53%), followed by good (33.76%) and general practice (7.34%).

**Table 5:** Practice dimension of the participants.

**Practice**	**Always *N* (%)**	**Often *N* (%)**	**Sometimes *N* (%)**	**Seldom *N* (%)**	**Never *N* (%)**
P1. You will actively popularize the knowledge of early gastrointestinal cancer to the patients	360 (66.06)	151 (27.71)	30 (5.50)	3 (0.55)	1 (0.18)
P2. You will actively participate in the popularization of knowledge on the importance of early gastrointestinal cancer screening as possible	220 (40.37)	149 (27.34)	136 (24.95)	33 (6.06)	7 (1.28)
P3. You will set yourself an example to your family and friends to develop a lifestyle that prevents the occurrence and progression of early gastrointestinal cancer	233 (42.75)	200 (36.70)	97 (17.80)	13 (2.39)	2 (0.37)
P4. You will actively introduce the prognosis of early gastrointestinal cancer to the diagnosed patients, and reduce the fear of patients to cancer	211 (38.72)	172 (31.56)	127 (23.30)	26 (4.77)	9 (1.65)
P5. You will actively introduce the treatments of early gastrointestinal cancer to the diagnosed patients, and alleviate the fear of patients to treatments	219 (40.18)	173 (31.74)	117 (21.47)	29 (5.32)	7 (1.28)
P6. You will actively warn the diagnosed patients to develop good lifestyles, and receive re-examinations regularly to prevent the development of early cancer	253 (46.42)	198 (36.33)	75 (13.76)	14 (2.57)	5 (0.92)

**Figure 1:**
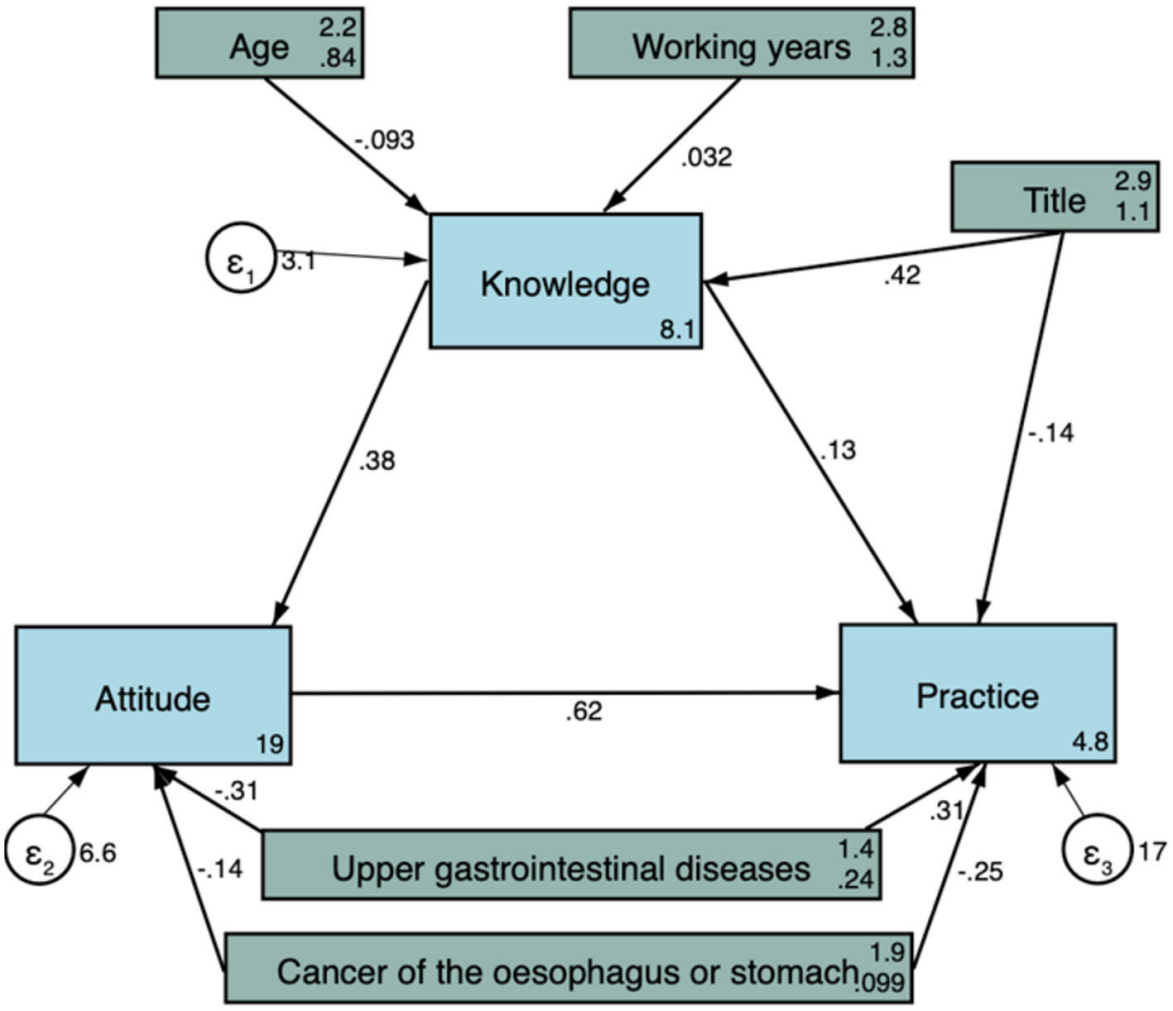
Structural equation model showing the associations between sociodemographic factors and KAP scores. All variables are observed variables. The direction of causality is indicated by single-headed arrows. The standardized path coefficients are presented alongside the arrows.

Pearson correlation analysis further revealed that knowledge score was positively correlated with both attitude (*r* = 0.264, *P* < 0.001) and practice score (*r* = 0.140, *P* = 0.001), and higher attitude score was significantly correlated with higher practice score (*r* = 0.380, *P* < 0.001) (Table 6). Moreover, the findings from the goodness-of-fit analysis indicated that the observed data aligned well with the proposed model, demonstrating consistency between the relationships among variables (Table 7). In addition, it was observed that professional title had a direct positive association with knowledge (path coefficient = 0.42, 95%CI: 0.21, 0.63, *P* < 0.001), while it had an indirect positive association with attitude (path coefficient = 0.16, 95%CI: 0.07, 0.26, *P* = 0.001) and practice score (path coefficient = 0.15, 95%CI: 0.04, 0.27, *P* = 0.010). Additionally, directly positive associations were found between knowledge and attitude (path coefficient = 0.38, 95%CI: 0.26, 0.50, *P* < 0.001), as well as between attitude and practice scores (path coefficient = 0.62, 95%CI: 0.48, 0.75, *P* < 0.001). In addition, a higher knowledge score was indirectly associated with higher practice score (path coefficient = 0.24, 95%CI: 0.14, 0.33, *P* < 0.001) (Table 8; Figure 1). These results from SEM supported and reinforced the main findings presented in the Pearson correlation analysis.

**Table 6:** Correlation analysis of KAP scores.

	**Knowledge**	**Attitude**	**Practice**
Knowledge	1		
Attitude	0.264 (*P <* 0.001)	1	
Practice	0.140 (*P =* 0.001)	0.380 (*P <* 0.001)	1

**Table 7:** Goodness of fit of SEM.

	**Value**	**Indicate**
RMSEA	0.030	Good fit
CFI	0.977	Good fit
TLI	0.940	Good fit
SRMR	0.021	Good fit

RMSEA, root mean square error of approximation; CFI, comparative fit index; TLI, Tucker–Lewis index; SRMR, standardized root mean square residual.

**Table 8:** The direct and indirect estimates of SEM.

**Model paths**	**Direct effect**		**Indirect effect**	

	β **(95% CI)**	***P***	β **(95% CI)**	***P***
Age → Knowledge	−0.09 (−0.40, 0.21)	0.545	-	-
Title → Knowledge	0.42 (0.21, 0.63)	<0.001	-	-
Working years → Knowledge	0.03 (−0.22, 0.29)	0.804	-	-
Knowledge → Attitude	0.38 (0.26, 0.50)	<0.001	-	-
Age → Attitude	-	-	−0.04 (−0.16, 0.08)	0.547
Upper gastrointestinal disease → Attitude	−0.31 (-0.75, 0.14)	0.174		
Title → Attitude	-	-	0.16 (0.07, 0.26)	0.001
Working years → Attitude	-	-	0.01 (−0.09, 0.11)	0.804
Esophageal or gastric cancer → Attitude	−0.14 (−0.83, 0.56)	0.702		
Knowledge → Practice	0.13 (−0.07, 0.33)	0.211	0.24 (0.14, 0.33)	<0.001
Attitude → Practice	0.62 (0.48, 0.75)	<0.001	-	-
Age → Practice	-	-	−0.03 (−0.15, 0.08)	0.551
Upper gastrointestinal disease → Practice	0.31 (−0.40, 1.02)	0.395	−0.19 (−0.47, 0.09)	0.179
Title → Practice	−0.14 (−0.49, 0.20)	0.411	0.15 (0.04, 0.27)	0.010
Working years → Practice	-	-	0.01 (−0.08, 0.11)	0.805
Esophageal or gastric cancer → Practice	−0.25 (−1.35, 0.85)	0.654	−0.08 (−0.51, 0.35)	0.702

## 4. Discussion

This study suggested that healthcare workers in China have moderate knowledge level, positive attitude, and excellent practice level on EGC. Good knowledge and positive attitude might be correlated with excellent practice. In addition, the KAP level might be influenced by sociodemographic characteristics. These findings might provide cues for hospitals about targeted educational intervention toward healthcare workers on EGC.

The high reliability of the questionnaire indicated consistent measurement of the underlying construct, and the adequate internal consistency suggested strong correlations among items within each construct. Furthermore, the results from Bartlett's test and KMO values supported the conduct of factor analysis, assessing the proportion of variance attributed to underlying factors. The robust construct validity observed confirmed the accurate measurement of intended constructs and was further supported by the factor analysis, revealing the underlying factor structure. These findings aligned with previous research emphasizing the importance of assessing reliability, internal consistency, and construct validity in questionnaire design (12, 13).

The participants obtained moderate knowledge of gastrointestinal cancer, who had a good understanding of cancer symptoms and prevention, but were unfamiliar with early-stage screening and treatment. Consistent with the present study, studies from Australia, China, Saudi Arabia, and Spain also corroborated the insufficient knowledge of gastrointestinal cancer screening among the population (14–17). In this study, we observed that the knowledge score would significantly increase or show an increment tendency as participants had advanced age, higher education level, superior professional titles, and long years of working. Similarly, Wong (18) found that lower educational attainment showed negative associations with knowledge of colorectal cancer screening. Alshammari and Alenazi (14) observed that respondents more than 50 years old had better knowledge regarding colorectal cancer. In addition, the association of mean knowledge rank with the physician's job title was also reported (19). These results could be interpreted that participants intended to obtain adequate knowledge of gastrointestinal cancer for the sake of an academic certificate or career promotion. However, Aldukhayel and Alsudairi (19) reported a negative association between knowledge and years of experience, which conflicted with our findings. Population heterogeneity, differences in socioeconomic background, and methodology diversity could account for the discrepancy. In addition, male healthcare workers scored better than their female counterparts in the knowledge dimension, which was in agreement with the finding of Demyati (20) but in conflict with the study of Mosli and Alnahdi (21). It could be attributed to the fact that common gastrointestinal cancers, such as gastric and colorectal cancer, were more prevalent among men; therefore, male participants were more likely to search and receive related knowledge (22, 23).

Compared with previous studies, the participants herein had higher attitude levels, especially in the propagation of knowledge on EGC (15, 17). As the screening of early-stage gastrointestinal cancer, such as gastroscopy, is carried out through opportunistic screening in China, mass participation is heavily influenced by individual self-consciousness (24, 25). Furthermore, participants' understanding of disease and action of taking hospital treatment are mainly impacted by healthcare providers; therefore, positive attitude toward gastrointestinal cancer among healthcare workers could help cancer prevention and control. We also observed that participants with years of working more than 5 years owned more positive attitudes compared with those with shorter years of working. In concordance with the study of Aldukhayel and Alsudairi (19), it supported the hypothesis that increased years of experience could contribute to overall awareness and correct attitude toward gastrointestinal cancer.

In accordance with the present study, more than half of the participants were with excellent practice scores in our study (26, 27). Most respondents were willing to popularize the information of EGC; however, reduced number of them were likely to propagate the early-stage screening. The above results could be explained by the inadequate knowledge of EGC screening among healthcare workers. Male healthcare workers were more active in the dissemination of gastrointestinal cancer prevention and treatment than female colleagues, partly due to higher knowledge scores among male subjects. Consistently, male subjects exhibited more enthusiasm for bowel cancer screening according to the research of Holden and Frank (28). However, Wang and Lin (29) reported that the female gender was positively associated with colorectal cancer screening. The aforementioned conflict could be derived from different sociodemographic characteristics, regional culture, and questionnaire design. Years of working were observed as an independent factor of practice score. It was understandable that expertise gets enhanced with the increment of years of working, which further exerts positive impacts on real-world practice. In addition, individuals with gastrointestinal discomfort tend to seek medical advice in the internal medicine department; therefore, healthcare workers in the internal medicine department could more frequently popularize gastrointestinal cancer and recommend screening.

It was noteworthy that knowledge was positively correlated with attitude, which was in agreement with previous literature (30, 31). As attitude included constituent of cognition, obtained knowledge helps formulate rational and lasting beliefs (32). Furthermore, subjects with higher knowledge and attitude scores were inclined to participate in practice more actively. Attitude helps to form judgment and evaluate the response to certain behavior; therefore, positive attitude could make practice more easily (33). Our results indicated that educational intervention could be of priority to target healthcare workers with female gender, <30 years old, lower education degree, private hospital, nurse occupation, junior job title, and shorter years of working.

This study first investigated the KAP status of EGC among healthcare workers in China and provided valuable information for future strategy formulation. In addition, several influential factors of KAP scores were identified, which facilitated targeted intervention. Based on the positive correlations among KAP scores, targeted educational intervention was proposed for the prevention and control of gastrointestinal cancer. However, several limitations also existed in our study. First, convenient sampling was adopted for participant collection, which could to some extent weaken the generalizability of results. Second, the sample size was relatively limited. Multi-center studies with larger sample sizes and higher response rates were needed to validate our findings.

In conclusion, healthcare workers in China have moderate knowledge level, positive attitude, and excellent practice level on EGC. Good knowledge and positive attitude might be correlated with excellent practice. KAP level might be influenced by sociodemographic characteristics. Targeted education could be further proposed to promote the overall knowledge, attitude, and practice of gastrointestinal cancer on EGC.

## Data availability statement

The original contributions presented in the study are included in the article/Supplementary material, further inquiries can be directed to the corresponding author.

## Ethics statement

The studies involving human participants were reviewed and approved by the Second People's Hospital of Lianyungang City in Jiangsu Province (# 2022016). The patients/participants provided their written informed consent to participate in this study.

## Author contributions

CZ and YW carried out the studies, participated in collecting data, and drafted the manuscript. DW and CS performed the statistical analysis and participated in its design. HZ and XL participated in the acquisition, analysis, or interpretation of data and draft the manuscript. All authors read and approved the final manuscript.
