# Supplementary material for: Knowledge, attitude, and practice of healthcare workers on early gastrointestinal cancer in China
Source: Front Public Health. 2023 Jul 6;11:1191699. doi: 10.3389/fpubh.2023.1191699 (PMC10357471; doi:10.3389/fpubh.2023.1191699)
Supplement: Supplementary file 1 [file Table_1.DOCX]

| Dear colleagues,  We are investigators from the Lianyungang second People’s Hospital, here we sincerely invite you to participate in this study that aims to assess the knowledge, attitude, and practice of medical personnel on early gastrointestinal cancer, and to provide evidence for developing scientific intervention strategies, which may help more people and improve their health in the future. Your participation in this study is voluntarily, and this study has been approved by an ethics review committee. If you agree to participate in this study, please read the instructions below.  1. Please complete the questionnaire, and there is no correct or wrong answer to the questions, thus please answer the questions according to your actual situation. Any questions encountered in answering the questions can be asked to us. Please submit the questionnaire in time after completion.  2. This study is a based on the simple questionnaire survey, which will not cause physical and psychological injuries. However, the study will involve some privacy issues, such as your sex, age, etc., The information will be kept strictly confidential and will not be disclosed, so please don’t worry about it when completing the questionnaire.  3. As a participant, you are entitled to learn the relevant information and study progresses. If you decide to withdraw from the study, please inform us in time, and you information will not be included in the analysis.  Finally, we sincerely thank you for making time to support this study.  □ I understand and consent that the data of mine collected in this study will be used for scientific investigation |
| --- |

| **The First Part** General characteristics | | | |
| --- | --- | --- | --- |
| 1. Your sex | a. Male | | b. Female |
| 2. Your age (years) | a. <30  b.31-40  c.41-50  d. >50 | | |
| 3. Your educational level | a. Technical secondary school/junior college  b. College  c. Master’s degree  d. Doctor’s degree | | |
| 4. Type of your hospital | a. Grade 1 public hospital  b. Grade 2 public hospital  c. Grade 3 public hospital  d. Private hospital | | |
| 5. Type of your occupation | a. Doctor  b. Nurse  c. Technician  d. Others | | |
| 6.Your professional title | a. None  b. Junior  c. Middle-grade  d. Associate chief  e. Chief | | |
| 7. Your working duration (years) | a. ≤5  b. 5-10  c. 11-15  d. ≥16 | | |
| 8. Your department：____________ | | | |
| 9. Have you, your family, or your friends been diagnosed with upper gastrointestinal disease such as chronic gastritis, reflux esophagitis, or gastric ulcer? | | a. Yes  b. No | |
| 10. Have your family been diagnosed with esophageal or gastric cancer? | | a. Yes  b. No | |

| **The Second Part** Knowledge of early gastrointestinal cancer | | | |  |
| --- | --- | --- | --- | --- |
| 1. The digestive tract includes the oral cavity, oropharynx, esophagus, stomach, duodenum, small intestine, colon, and rectum. | A. True | B. False | C. Not clear | |
| 2. Early esophageal cancer refers to esophageal cancer with invasion depth of reaching the mucosal layer, but not accompanied with lymph node metastasis. | A. True | B. False | C. Not clear | |
| 3. Early gastric cancer refers to the tumors that the invasion limited to the mucosal or submucosal layer, and is related to the lesion size and lymph node metastasis. | A. True | B. False | C. Not clear | |
| 4. Early colorectal cancer refers to the disease that the cancerous tissue restricted to the mucosal and submucosal layer, regardless of lymph node metastasis. | A. True | B. False | C. Not clear | |
| 5. Early gastrointestinal cancer refers to early tumors of the digestive tract; most digestive tract cancers have no specific symptoms in the early stage and thus are easy to be ignored. | A. True | B. False | C. Not clear | |
| 6. Individuals aged >40 years old, with the history of precancerous lesions of the digestive tract, family history of digestive tract tumor, evident gastrointestinal symptoms, prefer salty, fried, or smoked food (> 3 meals/week), and/or heavy smoking and alcohol drinking are considered high-risk population. | A. True | B. False | C. Not clear | |
| 7. The first and foremost task of early gastrointestinal cancer screening is to identify the high-risk population. | A. True | B. False | C. Not clear | |
| 8. Currently, tumor marker detection, such as CEA and CA125, is the major screening method. | A. True | B. False | C. Not clear | |
| 9. Endoscopy can identify the lesions in the digestive tract, but cannot obtain biopsy of the cancerous sites for pathological examination. | A. True | B. False | C. Not clear | |
| 10. Early gastrointestinal cancer can be completely removed under endoscopy, while no chemotherapy is required after surgery, and the 5-year survival rate is > 90%. | A. True | B. False | C. Not clear | |
| 11. Compared with regular endoscopy, precision endoscopy can provide the examinations of magnifying endoscopy, staining endoscopy, and electronic staining endoscopy, which makes the lesion examination more subtle, therefore help determining whether the lesion is cancerous, as well as the range and depth of invasion, and differentiation degree, and evaluate the presence of indications for microscopic treatment. | A. True | B. False | C. Not clear | |
| 12. Compared with traditional endoscopy, the emerging endoscopic narrow band imaging (NBI) in recent years allows us not only to accurately observe the morphology of the mucosal epithelium of the digestive tract, but also observe the morphology of the epithelial vascular network, and thereby improving the accuracy of endoscopic diagnosis. | A. True | B. False | C. Not clear | |
| 13. Early gastrointestinal cancer is mainly treated endoscopically, including endoscopic mucosal resection (EMR) and endoscopic submucosal dissection (ESD), of which EMR is more frequently used. EMR can be used for patients with early stage of esophageal cancer, gastric cancer, or colorectal cancer. | A. True | B. False | C. Not clear | |
| 14. To prevent early gastrointestinal cancer, the following items should be paid with additional attentions in daily living: appropriate dietary structure, personal hygiene, regularly diet, avoiding heavy smoking or drinking, and defecate every day. | A. True | B. False | C. Not clear | |

| **The Third Part** Attitude to early gastrointestinal cancer | | | | | |
| --- | --- | --- | --- | --- | --- |
| 1. You think high-risk population, regardless of whether they have symptoms or not, should receive the screen for early gastrointestinal cancer. | a. Highly agree | b. Agree | c. Neutral | d. Disagree | e. Highly disagree |
| 3. You think that early esophageal cancer and stomach cancer can be radically treated (completely cured). | a. Highly agree | b. Agree | c. Neutral | d. Disagree | e. Highly disagree |
| 4. You think that changing the lifestyle can prevent the occurrence of digestive tract cancer. | a. Highly agree | b. Agree | c. Neutral | d. Disagree | e. Highly disagree |
| 5. You think that the general people's awareness of early gastrointestinal cancer is far from enough, and it is necessary to increase the intensity of health education. | a. Highly agree | b. Agree | c. Neutral | d. Disagree | e. Highly disagree |
| 6. You think that popularization of science in general people is of great significance for the reduction of the occurrence and development of early gastrointestinal cancer. | a. Highly agree | b. Agree | c. Neutral | d. Disagree | e. Highly disagree |
| 7. You are willing to do your best to popularize the knowledge of early gastrointestinal cancer. | a. Highly agree | b. Agree | c. Neutral | d. Disagree | e. Highly disagree |
| 8. Which of the followings do you consider to be risk factors of gastrointestinal cancer? 0 indicates no or unclear, and 1 indicates yes. Please write down the corresponding numbers according to your own understanding. (This question is not included in the analysis of reliability and validity).  Advanced age:____;  Male____;  Family history of esophageal or gastric cancer (parents or siblings have esophageal or gastric cancer) ____;  Long-term smoking (>5 cigarette/d on average, and the habit has lasted for several years) ____;  Long-term heavy alcohol drinking (>150 g/d for males, or >100 g/d for females on average for more than 5 years) ____;  High-salt diet (salt intake >10 g/d on average (equal to the salt of about one cap of a bottle of mineral water) ____;  Spicy diet____;  Oily diet____;  Eat pickled, smoked, fried, or deep fried food often (≥2 times/week on average) ____;  Eat vegetables or fruits often (≥400 g/d on average) ____;  Eat processed meat or sausages often (≥2 times/week on average) ____;  Eat leftovers often (especially leftovers of the previous day) (≥2 times/week on average) ____;  Usually eat hot meal and drink hot water or hot tea ____;  Irregular diet, such as no definite for three meals, each extra meal at midnight, or not eating breakfast (2 times/week on average)____;  Eat very fast (time for finishing a meal <10 min on average) ____;  Overeating____;  Obesity (body-mass index≥30) ____;  Lack of exercise (moderate exercises, such as fast walking or riding a bike for <30 min/time, <3 d/week) ____;  With fast pace of life and being stressful ____;  Being sulking and in depressive mood often ____;  Air pollution____;  Helicobacter pylori infection ____;  Chronic esophagitis ____;  Gastroesophageal reflux disease ____;  Esophageal ulcer ____;  Chronic gastritis ____;  Gastric ulcer ____;  Gastric polyp ____; | | | | | |

| **The Fourth Part** Practice on early gastrointestinal cancer | | | | | |
| --- | --- | --- | --- | --- | --- |
| 1. You will actively popularize the knowledge of early gastrointestinal cancer to the patients. | a. Always | b. Often | c. Sometimes | d. Seldom | e. Never |
| 2. You will actively participate in the popularization of knowledge on the importance of early gastrointestinal cancer screening as possible. | a. Always | b. Often | c. Sometimes | d. Seldom | e. Never |
| 3. You will set yourself an example to your family and friends to develop a lifestyle that prevents the occurrence and progression of early gastrointestinal cancer. | a. Always | b. Often | c. Sometimes | d. Seldom | e. Never |
| 4. You will actively introduce the prognosis of early gastrointestinal cancer to the diagnosed patients, and reduce the fear of patients to cancer. | a. Always | b. Often | c. Sometimes | d. Seldom | e. Never |
| 5. You will actively introduce the treatments of early gastrointestinal cancer to the diagnosed patients, and alleviate the fear of patients to treatments. | a. Always | b. Often | c. Sometimes | d. Seldom | e. Never |
| 6. You will actively warn the diagnosed patients to develop good lifestyle, and receive re-examinations regularly to prevent the development of early cancer. | a. Always | b. Often | c. Sometimes | d. Seldom | e. Never |
